# Pulsed-Field Gel Electrophoresis Analysis of Bovine Associated *Staphylococcus aureus*: A Review

**DOI:** 10.3390/pathogens12070966

**Published:** 2023-07-24

**Authors:** Zoubida Dendani Chadi, Marie-Anne Arcangioli

**Affiliations:** 1 Laboratory of Biodiversity and Pollution of Ecosystems, Department of Veterinary Medicine, Faculty of Natural Science and Life, University of Chadli Bendjedid, El Tarf 36000, Algeria; 2 VetAgro Sup, Université de Lyon, UMR Mycoplasmoses Animales, 69280 Marcy l’Etoile, France; marie-anne.arcangioli@vetagro-sup.fr

**Keywords:** cattle, contour-clamped homogeneous electric field (CHEF), gel electrophoresis, methicillin-resistant *Staphylococcus aureus* (MRSA), outbreaks, pulsed-field gel electrophoresis (PFGE), *Staphylococcus aureus* (*S. aureus*)

## Abstract

For decades now, DNA fingerprinting by means of pulsed-field gel electrophoresis (PFGE) continues to be the most widely used to separate large DNA molecules and distinguish between different strains in alternating pulses. This is done by isolating intact chromosomal DNA and using restriction enzymes with specific restriction sites to generate less than 30 restriction fragments from 50 Kb to 10 Mbp. These results make clone-specific band profiles easy to compare. Specialized equipment is required for the optimization of DNA separation and resolution, among which a contour-clamped homogeneous electric field (CHEF) apparatus is the most commonly used. As a result, the PFGE analysis of a bacterial genome provides useful information in terms of epidemiological investigations of different bacterial pathogens. For *Staphylococcus aureus* subtyping, despite its limitations and the emergence of alternative methods, PFGE analysis has proven to be an adequate choice and the gold standard for determining genetic relatedness, especially in outbreak detection and short-term surveillance in the veterinary field.

## 1. Introduction

*Staphylococcus aureus* (*S. aureus*), from the coagulase-positive staphylococci (CoPS) group, is a major human pathogen and a major cause of bovine intramammary infections (IMIs) throughout the world [1]. In humans, *S. aureus* is being increasingly recognized as virulent and is associated with nosocomial and community-associated infections. In addition, it is the third emergent zoonotic pathogen worldwide [2,3], since, by causing IMIs in cattle, *S. aureus* contaminates milk and other dairy products, causes foodborne illness, produces diverse *Staphylococcus* enterotoxins (SEs) [4], and represents a public health risk [5,6,7]. In Europe, SEs are the third principal cause of food poisoning outbreaks (FPOs) [8]. There are 28 SEs and enterotoxin-like (SEl-) toxins that have been identified, including the classical and the newer types [9]. According to Wang et al. [10], more than 90% of FPOs that are related to *S. aureus* are linked to the SEs, particularly SEA to SEE, and are encoded by *sea* to *see* genes. All of them are encoded on different chromosomal pathogenicity islands [11]. Each of the classical enterotoxins has been shown to be responsible for outbreaks due to raw milk consumption [12,13]. Most of the SEs (designated SEG–SEQ) also occur in *S. aureus* strains isolated from bovine IMIs [14]. Bovine mastitis is the most frequent and costly infectious disease occurring in dairy cows, impacting milk production and leading to economic losses. *S. aureus,* which is frequently connected with clinical and subclinical outbreaks of mastitis [15], is considered a contagious pathogen by means of the mammary gland through the teat canal [16]. Extramammary sites, including the milking parlor, milking utensils, cooling equipment, and milker’s hands, are also common means of transmission and the persistence of *S. aureus* [17,18]. This bacterium is of growing concern, as it is related to an increased use of antibiotics in animal production and to the resulting development of antibiotic resistance risk [19]. Therefore, *S. aureus* has become methicillin-resistant (MRSA) by acquiring the *mec*A gene, a major drug-resistant gene, by a cassette situated in the chromosome (SCC*mec*). Three main types of MRSA have evolved: the first “healthcare-associated MRSA (HA-MRSA)” dating back to the 1960s, the second “community-associated MRSA (CA MRSA)” starting in the 1990s, and the third “livestock-associated MRSA (LA-MRSA)” emerged during the 2000s [19,20]. This third “wave” of MRSA emergence, especially in cattle, confirms that new reservoirs for MRSA exist [7]. When humans and/or animals are in close contact with each other [20], host switching may occur [21]. These strains share some identical genes, and they are becoming a major health issue for both animals and the public health [22]. To understand the genetic background of the drug-resistant pathogen and to determine appropriate preventive advice and actions, the clonal diversity in *S. aureus* isolates should be detected and evaluated [6,10,23]. Whole genomic approaches can meet this requirement, as they are reproductible and display an excellent discriminatory power [24]. Some common molecular methods can be used, such as pulsed-field gel electrophoresis (PFGE), multiple PCRs, multi-locus sequence typing (MLST), staphylococcal protein A (*spa*), and Staphylococcal cassette chromosome *mec* (SCC*mec*) typing [25]. PFGE has been in use for the past three decades and is still the gold standard fingerprinting method for *S. aureus* subtyping [10,19]. Due to its multidirectional migration schemes, this method continues to be the most widely used in separating large fragments of deoxyribonucleic acid (DNA) molecules from intact bacterial chromosomes. In this review, an overview of the literature on PFGE analysis is presented. The current knowledge on the development, principles, and practical aspects of PFGE analysis are summarized, highlighting its specific advantages and disadvantages. Its contribution to different applications in the epidemiologic investigation of bovine *S. aureus* is discussed and evaluated in comparison with the main molecular typing methods for *S. aureus*, such as MLST, *spa*, and SCC*mec* typing [25], as well as newer techniques, such as whole genome sequencing (WGS) and DNA microarrays [26].

## 2. The Evolution of PFGE Approaches

### 2.1. Brief History of Standard Gel Electrophoresis

Electrophoresis has been extensively employed in biological science research to separate macromolecules, such as DNA, RNA, and proteins, by size and charge [27]. The study of DNA electrophoresis began in 1964 and was developed in the 1970s [27]. To separate DNA, agarose gel electrophoresis has proven to be an easy and efficient method that is now widely used after either restriction digestion or PCR amplification. Small molecules, 100 bp to 25 kb in size, submitted to a uniform electric field migrate in an agarose gel. They are separated on the basis of their size and charge and are usually detected by ethidium bromide staining and exposure to ultraviolet (UV) light [28]. The movement is faster in small fragments compared to large ones. Agarose gel electrophoresis has been shown to be an efficient and effective tool for separating DNA fragments, as it is easy to perform, rapid, and appropriate for most laboratories, leading to genome-based epidemiological analysis [16]. However, electrophoresis is hindered by the limitations of continuous field agarose gel, which is unable to separate DNA fragments that are larger than 30–50 kb [29,30] and to discriminate accurately those larger fragments. Subsequently, the gel loses its screening action, and the fragments take the form of a large non-resolving band with an abnormally excessive degree of mobility [30]. During electrophoresis, the separation and mobility of large DNA fragments are affected by various factors, such as the gel’s composition and concentration, buffer used, temperature, and the voltage gradient of the electric field [30].

### 2.2. Principles of PFGE

In 1984, Schwartz and Cantor [31] depicted an application of an alternating current from different directions, which they named pulsed-field gel electrophoresis (PFGE), as an alternative approach for the electrophoresis of large fragments, with the separation of intact yeast chromosome DNA. In contrast to standard electrophoresis, PFGE has the ability to separate molecules as large as 10 Mbp [32]. The direction of the electric field is constantly changed, and the DNA molecules are reoriented through the agarose gel following the same rate of change [29]. Larger DNAs align their charge more slowly to the change in direction, but smaller DNAs are quicker, resulting in greater separation [30]. Therefore, DNA molecule separation using PFGE largely depends on the expected time for each current direction [33]. Since that time, the use of PFGE has been expanded to cover several bacteria’s molecular epidemiology, including *S. aureus*. All PFGE methods are based on the lysis of bacterial cells after integration into agarose gel molds to gently cut the chromosomal DNA using rare cutting endonucleases for the separation of large DNA fragments [32]. Several studies have applied PFGE using a variety of restriction enzymes, including *Sma*I, *Csp*I, *Sst*II, *Sgr*AI, *Xba*I, and *Cf9*I [29,34,35]. *Sma*I is the most used enzyme and is the best choice for genotyping *S. aureus* isolates [36], yielding between 20 and 30 molecular weight DNA fragments after digestion [33,37]. This technique is adequate for entire bacterial genome representation, providing well-defined and highly resolved fragments of DNA. The resulting genetic fingerprint of pathogens is reproducible [30]. The different DNA bands obtained on the agarose gel are referred to as the “DNA fingerprint”. They are subsequently utilized for distinguishing clonal relationships between strains [38], depending on the number and position of restriction sites in the genome. The main advance made in developing PFGE was the production of a homogeneous or a variable electric field using multiple electrodes and different voltages and directions [30].

### 2.3. PFGE Instruments

Since PFGE technology began to evolve, many kinds of PFGE tools have been created. These differ by the direction of the applied current [39,40,41], including the contour-clamped homogeneous electric field (CHEF) [42], transverse alternating field electrophoresis (TAFE) [43], orthogonal field gel electrophoresis (OFAGE) [44], rotating gel electrophoresis (RGE) [45], field inversion gel electrophoresis (FIGE) [46], and programmable autonomously controlled electrodes (PACE) [47]. The different types of PFGE devices and their main traits are summarized in Table 1. In general, all these PFGE instruments use multiple electric fields, separate the same size range of DNA, and produce uniform migration. However, they differ in terms of electrode configuration, the angle of reorientation, the speed of separation, and the resolution obtained in any specific size range [47]. The choice of the PFGE tool is primarily based on two factors: PFGE performance and its balance against the instrument’s cost. CHEF electrophoresis is the most widely used variant of electrophoresis in pulsed fields today, as it can use 24 electrodes and successfully resolve molecules’ range from 10 kbp to the larger 10 Mbp [47,48]. This apparatus, which generates straight lanes and stable DNA separation, was developed by Chu et al. [42] for the resolution of whole bacterial chromosomes. Migrating DNA fragments are made to move by periodic pulses of an electric field that is applied in two alternating directions through the gel over an angle of 120° [29], both of which are homogeneous along the gel’s width and length [48]. In such a system, different aspects of the electric field, such as size, location, coordination, stability, and continuity, are subjected to precise control [41]. A CHEF apparatus is easy to use, able to separate many DNA samples, and able to produce straight lanes easy to compare to each other [48]. In addition, CHEF has become the optimal system for epidemiological studies [42] and is so widely used in laboratories that it has generally replaced other PFGEs, noticeably for the highly standardized Pulse Net International Network (www.pulsenetinternational.org, accessed on 30 August 2019).

### 2.4. PFGE Analysis

**Figure 1:**
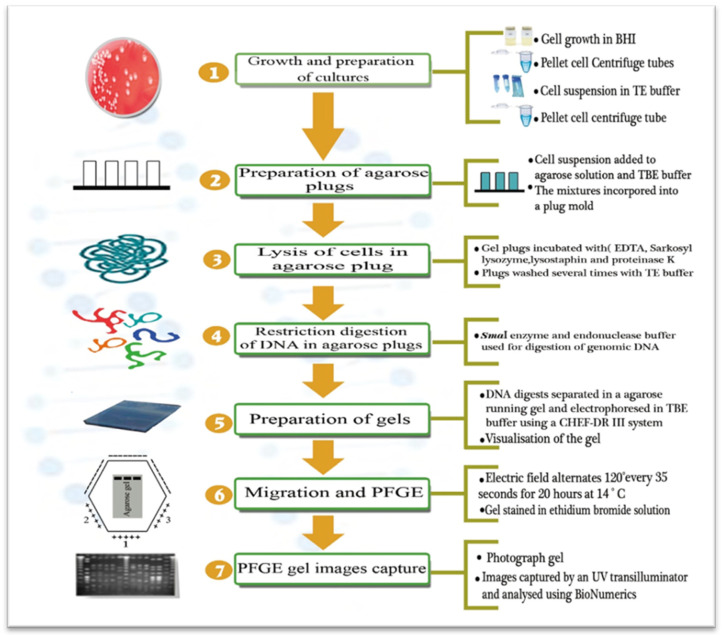
PFGE process for the subtyping of *S. aureus* strains.

This procedure describes PFGE, which is a method developed to reveal the genomic relatedness among *S. aureus* strains and to identify potential sources of infection. PFGE protocols generally take 3 to 4 days, excluding the isolation and growth of bacteria cultures. The agarose plugs and process of lysis of the cells, as well as restriction enzyme digestion and the electrophoresis processes, were primarily described by Talon et al. [49] and then modified and adapted to *S. aureus* by Zschöck et al. [50] and Dendani Chadi et al. [51]. The lysing methods, restriction enzymes, and electrophoresis run times may vary among PFGE protocols. The main steps of subtyping *S. aureus* strains using PFGE are shown in Figure 1. The important stage is the lysis of the cells into an agarose plug to avoid the mechanical cutting of DNA.

#### 2.4.1. Strains Preparation for PFGE Analysis

To prepare DNA, the strains must be isolated and cultured overnight at 37 °C in 5% bovine blood agar. *S. aureus* cells from the overnight cultures are harvested and incubated in 5 mL of brain heart infusion broth at 37 °C for 18 h. These cell cultures from overnight are harvested and followed by 2 h of incubation at 37 °C in a similar broth [51].

#### 2.4.2. Preparation of the Agarose Plugs and Lysis of the Cells

The particularity of this method lies in the isolation of the DNA after embedding in agarose plugs [48]. From each culture, an aliquot of 1 mL of cell culture is centrifuged at 12,000× *g* and rinsed with 1 mL of 1 × TN buffer (10 mM Tris-HCl, pH 8.0, 1 M NaCl). The cells are centrifuged again at 12,000× *g*, suspended in 150 μL of the same buffer, and maintained at 42 °C. To prepare the agarose plugs, the cell suspension is added to an equal volume of 1.6% (*w*/*v*) agarose solution. The mix of cells and agarose is poured into a mold for plug formation and set to solidify at room temperature for 30 min. After solidification, the gel plugs are placed into a tube with 1.5 mL of 1 × Lysis buffer (0.5 M EDTA, pH 8.0, 1% Sarkosyl), to which 30 μL of lysostaphin solution (5 mg/mL) and 15 mg of lysozyme are added. The tube is incubated in a water bath at 55 °C for 1 h. A second and complementary lysis with proteinase K solution (1%) is then done for 15 min, and then they are rinsed with 5 mL of TE buffer at least four times at 37 °C for 1 h each time before 2 mL are stored at 4 °C.

#### 2.4.3. Restriction Digestion in Agarose Plugs

This step generally takes 2 days. For DNA digestion, the plugs are cut into small slices. Restriction enzyme digestion is carried out using 300 μL solution containing 30 μL of the appropriate 10 × restriction buffer, 3 μL bovine serum albumin (BSA), and sterile water and incubated overnight at 4 °C. For the discrimination of the *S. aureus* species, the plugs are digested with 3 μL (30 U) of *Sma*I restriction enzyme and incubated at 25 °C. This solution is pulled out, and the plugs with digested DNA are rinsed in 500 μL of TE buffer (10 mM Tris-HCl, 1 mM EDTA (pH 8.0)).

#### 2.4.4. Electrophoresis Conditions

After restriction cutting, the plugs are loaded into wells of a 1% agarose running gel. After solidification at room temperature, the gel is placed in the apparatus and bathed in a running buffer containing 2 L of 0.5 × TBE. For our experience, electrophoresis was performed in a CHEF DR-III system (Bio-Rad Laboratories) under the following conditions: 20 h of running at 14 °C with an initial and final switching time of 5 s and 35 s, an angle of deviation of 120°, and a voltage of 6 V/cm or 200 V. A lambda ladder was used as the molecular weight standard and *S. aureus* ATCC 29213 as the reference strain. After running, the gels were stained in 15 μL of this UV dye (ethidium bromide, for example) for 10 min, destained for 20 min in distilled water, and visualized under ultraviolet transillumination.

### 2.5. Interpretation of PFGE

The results can be interpreted both visually or by computer-assisted analysis after photographed with a camera. For the comparison of a few isolates, the visual analysis of the banding patterns of one gel is easy. The criteria developed by Tenover et al. [52] are applied for assessing *S. aureus* strains’ genetic relatedness. Strains are considered clones when the numbers and positions of the bands are 100% similar and genetically indistinguishable. Strains classified as closely related differed by no more than three bands in their patterns, which may have arisen from only one genetic event. Strains with four or more different bands are considered unrelated. The similarity coefficient is set to 80%, as recommended by Struelens et al. [53]. Patterns may be compared using comparison software to calculate the Dice coefficients of correlation and to construct dendrograms of similarity [54].

## 3. Advantages

Many genome-based techniques have been developed and applied to different epidemiological and genetic purposes in relation to various bacteria, among which PFGE is widely used. Such a method is a useful starting point for more detailed analyses since the whole chromosome is separated, and large fragments of DNA are generated [29,30]. The introduction of PFGE in the 1980s has made it possible to study and map the entire genome of bacteria. Capillary gel electrophoresis 10 years after has rendered it possible to sequence those entire chromosomes. The success of PFGE results from its excellent discriminatory power [55] and concordance with epidemiological relatedness and no need for sequence information [15]. It is the gold standard for short-term surveys of mastitis outbreaks [54,56] and surveillance in foodborne diseases linked to *S. aureus* [57,58,59]. Furthermore, PFGE is applicable in a broad range of bacterial species; it is less expensive and easier to perform than some highly discriminatory methods, such as whole genome sequencing (WGS), since it does not require detailed biological information or software [48,60]. PFGE’s discriminatory power and reproducibility tend to be higher than that of multiple-locus variable number tandem repeat analysis (MLVA), staphylococcal protein A (*spa*), multi-locus sequence typing (MLST), ribotyping, restriction fragment length polymorphism of the coagulase (PCR-RFLP) of the *coa* gene, phage typing, amplified fragment length polymorphism (AFLP), and ribosomal spacer (RS-PCR) in characterizing *S. aureus*, including MRSA strains [19,61]. Additionally, owing to its highly discriminative characteristics, PFGE analysis is now relied upon by most laboratories and research centers [30]. For example, many countries, such as Canada; the USA; China; and other countries in Africa, Europe, Latin America, and the Caribbean, have established a nomenclature for their *S. aureus* local pulsotypes to monitor foodborne diseases through the standardization of PFGE protocols with the use of PulseNet (www.pulsenetinternational.org, accessed on 30 August 2019) [32,40,62,63]. This system makes the comparison of the investigated strains’ PFGE profiles with those available in the database easier, and it determines the relatedness of strains to those found in an outbreak [32].

## 4. Limitations

Despite the various merits of PFGE analysis, drawbacks remain, and the reasons for these are manifold. First, a significant limitation of PFGE is that it can be performed only in well-equipped laboratories. Second, PFGE is time-consuming, as most of the protocols require more than 4 days to complete [64,65], which is really too long when some profiles are impossible to interpret, especially in the case of large collections of isolates [51]. MRSA isolates of ST398 have been found to be non-typeable by the *Sma*I restriction enzyme [66], since no banding patterns were generated [10,55]. But they were able to be digested with *Cfr9*I, a neoschizomer of *Sma*I [67]. If interlaboratory results are comparable, a comprehensive pooling of profiles in an international profile bank is necessary for that [15]. What is more, PFGE is not the optimal method for long-term epidemiological surveillance or phylogenetic relationship evolution in *S. aureus* strains [32], as modest variations in a restriction site are enough to generate large differences in profiles, masking possible epidemiological links, a limit to sufficient stability [34,68].

Most importantly, DNA fingerprinting by PFGE has serious limitations when used to investigate the finer details of infection outbreaks [69]. PFGE lacks sufficient resolution to distinguish nearly identical bands that differ in size by <5% and smaller fragments of less than 20.5 kb [70]. It is therefore difficult to infer phylogeny from PFGE data [70,71]. This could be explained by the fact that some changes occur in the PFGE pattern, as losing or gaining chromosomal mobile genetic elements (MGEs) can alter the clone-specific banding pattern [68]. Since the relationship between isolates is inferred by the similarities of the restriction fragment pattern, this leads to different fragment patterns in the PFGE results and frequent errors in band assignments [65]. Therefore, the different fragment patterns of two independent isolates do not always exclude a common source. To address these issues, it was recommended that, first, PFGE patterns must always be interpreted in the context of the proper epidemiological and clinical characteristics of isolates, since, in the absence of epidemiologic information, strains with different PFGE patterns may be epidemiologically related [32]. Second, the choice of the restriction enzyme and conditions for electrophoresis need to be optimized. More restriction enzymes would be needed for a reasonable PFGE estimation, since it has been shown that single-enzyme DNA macrorestriction profiles differing by up to three fragments are more likely to represent genotypic variants of the same epidemic MRSA clone. Indeed, a single-point mutation in the bacterial chromosome can introduce such a three-fragment difference in a restriction pattern [53].

## 5. Advances in the Use of PFGE in Epidemiologic Studies of Bovine *S. aureus*

Among the plethora of genome-based techniques, PFGE uses restriction endonucleases to separate the *S. aureus* genome into a few large fragments. Generated band patterns are convenient for chromosomal fingerprinting and physical mapping, as well as for determining the level of the relationship between strains [72]. For this aim, PFGE analysis has been the most widely used approach for decades [48], with significant applications, such as in phylogenetic diversity, clonal relatedness, outbreak detection, and surveillance [38,73,74]. Studies of genetic diversity have provided information on the clonal relationship between strains, the context of the outbreak setting, and the source of infections [15]. In addition to aiding in mastitis surveillance programs, both within and between herds [10], strain identification seems at least partly informative in terms of genetic diversity and the effects on milk production [75].

### 5.1. Genetic Diversity of Bovine S. aureus Strains

*S. aureus* is among the most studied bacteria in bovine mastitis [1]. The first staphylococcal genomes sequenced to completion belonged to colonizing and pathogenic species of *S. aureus* [76]. In cattle, since the first genome of *S. aureus* strain RF122 was reported by Herron-Olson et al. [77], several population studies undertaken by genomic means have been published [1]. This pathogen’s genome size is between 2.6 and 3.1 Mb, with an average GC content of 32.8% [76,78]. Staphylococcal genomes are composed of the core and accessory components. The former, which encodes genes present in all isolates, comprises approximately 75% of the 2.8 Mbp genome. The latter, which represents 25% of the total genome, is dominated by mobile genetic elements (MGEs), the main agents of horizontal gene transfer [79]. Many types of MGEs are found in bovine *S. aureus*. Bacteriophages, *S. aureus* pathogenicity islands (SaPIs), transposons, and plasmids have been identified as carriers of virulence factors, including Staphylococcal enterotoxins (SEs) genes, while chromosome cassettes (SCC*mec*) carry the methicillin resistance gene [68,80]. The antibiotic resistance genes might be disseminated and transferred horizontally through their emergence within antimicrobial-resistant *Staphylococci* [81]. The acquisition or loss of MGEs and diversification in their genetic content are responsible for the host specificities observed among *S. aureus* clones [1,82] and the great diversity of strains, as *S. aureus* varies in pathogenicity upon the acquisition of new genetic elements [68,76]. Comparative genomic analyses have revealed that pathogenic clones of *S. aureus* contain extensive genetic variations of the genome content, particularly in relation to MGEs [76]. All these phenomena play an important role in bacterial evolution [55]. In evaluating the DNA diversity, most PFGE-based reports have shown high genetic heterogeneity between *S. aureus* isolates obtained from dairy cows with mastitis, illustrating the independence of lineage evolution worldwide [15,23,35,36,59,83,84,85,86,87,88,89]. In contrast, some others have shown limited genetic diversity among isolates within and between herds [90,91,92,93,94]. Those results are consistent with the contagious nature of *S. aureus* by horizontal spreading between cows in close milking contact. The wide variety of these findings reflects the differences in *S. aureus* and MRSA populations worldwide, as demonstrated by the variability in the degree of similarities between isolates with the following rates reported: Poland (44.8%) [13], Sweden (100%) [86], France (57.5%) [95], Finland (80%) [82], Czech Republic (88%) [55], India (80%) [15], Brazil (80%) [89], the USA (80%) [90], South Africa (80%) [96], China (92%) [10], the Republic of Korea (85%) [97], Italy (90–100%) [98], Hungary (86%) [91], Niger (60–94%) [99], and Turkey (88–90%) [100]. The great diversity of genotypes may arise from the fact that no endemic clone predominates in the environment [84]. It might also be explained by the variation in the geographic areas and origins of isolates [12] or by the intense animal traffic and diversity of locations in which *S. aureus* may be found [17]. According to Alibayov et al. [55] and Korpysa-Dzirba and Osek [13], the high diversity of this bacteria indicates that contamination can come from several sources, and according to Middleton et al. [101] and Haveri et al. [82], the likelihood of its increase is related to the introduction of new strains through animal imports. Various management practices are thought to influence genetic diversity, as displayed by Issa et al. [99], Oliveira et al. [94], and Schmidt et al. [96]. Limited genetic diversity is an indication of transmission and circulation of the same clones [96,102]. Their similarity can also be explained by the possible purchasing of products from the same supplier at different locations or common milking machines within herds [93] or the persistence of strains that are unlikely to undergo any major genetic changes [87,103,104]. In the end, many results have shown that *S. aureus* strains harbor more than one genotype, but a small number of host-specialized clones predominate in bovine IMIs [70,99].

### 5.2. Genotypic Relatedness and Outbreak of Bovine S. aureus

Outbreaks are viewed as short-term events or cases of local epidemy. The organisms involved in outbreaks are genetically identical or clonally related. However, at the time of an outbreak, strains capable of horizontal gene transfer are susceptible to genetic changes [65]. In the setting of a suspected outbreak, it is necessary to stop disease spreading and prevent additional cases. Understanding how strains are linked aids in the comprehension of how such outbreaks occur [105]. Genomic surveillance can detect whether these outbreaks are caused by the same strain, and it can monitor their emergence and spread within human or veterinary medicine [25,105]. In fact, considerable investigations into *S. aureus’* genetic relatedness have been done in the context of outbreak IMIs using a variety of methods, such as DNA sequence-based (MLST, *spa* typing) [106]; PCR-based typing techniques such as RAPD [107], RS-PCR [108], PCR-RFLP [109], and SCC*mec* typing [98]; multi-locus enzyme electrophoresis (MLEE) and ribotyping [110] and AFLP [111]; PFGE typing [90,112]; MLVA [113]; and, more recently, WGS [24] and DNA microarrays [58,114].

#### 5.2.1. The Comparison of PFGE Analysis with the Main Molecular Typing Methods for *S. aureus* Isolated from Bovine IMIs

The utility of these typing systems depends on the nature of the investigation for which they are used. They differ from one another in cost, ease of use, and discriminatory power (Table 2). Comparing some of these molecular methods has allowed an evaluation of the relative strengths and weaknesses of each method. MLVA appears to be the most useful PCR-based method for its high-throughput [113], low cost, ease of use, and ability to provide rapid results but is significantly less discriminatory than PFGE techniques [19]. MLVA is favorable for determining genetic diversity, the evolution and emergence of host- or udder-adapted clones [19,113], and with PFGE, MLST, and *spa* typing, it is currently used for local and international large-scale analyses of the molecular epidemiology of *S. aureus* and MRSA isolates involved in bovine mastitis [19,113]. Sequence-based methods, such as MLST and *spa* typing, are the most powerful tools for large-scale analyses of diverse *S. aureus* isolates and the clonal evolution of MRSA due to their standardized nomenclature and portability into an international database [19]. Although MLST with seven housekeeping genes is considered the gold standard in terms of phylogenetic relationships among strains, it is less useful for outbreak settings, as it has limited discriminatory power and is more costly than PFGE [96]. *spa* gene typing, which is based on the protein A-coding gene, is the most widely used for establishing clonal relationships between *S. aureus* strains [6], investigating MRSA outbreaks, and epidemiological surveillance due to its simplicity, relatively low cost, and high-throughput [71]. However, it is not only less discriminatory than PFGE, especially for the characterization of *S. aureus* food-associated isolates [64,108,114], but it only considers a very limited portion of the whole genome and has limited resolution [115]. SCC*mec* elements are mobile sequences comprising the *mec* gene complex (*mec*), which is responsible for the resistance to methicillin, and the *ccr* gene complex (*ccr*), which is charged with the integration and excision of the cassette in the bacterial genome [70]. *SCCmec* has become popular for epidemiologic and evolutionary analyses of LA-MRSA strains [19,70], which play a core role in antimicrobial resistance characteristics. SCC*mec* can detect the type of SCC*mec* cassette but not its structure. It takes time and is complex, since the SCC*mec* region is variable, and new types are constantly being defined, and it is less discriminatory than PFGE for strain relationships [19]. The majority of these techniques are not sufficiently discriminatory or useful in an outbreak to be acceptable, since they produce inconclusive results due to poor resolution [69]. PFGE has been promoted as the gold standard, since it is highly discriminatory and it performs well in the context of a local outbreak. However, in terms of discriminatory power and resolution, WGS and next-generation sequencing (NGS) outperform all other methods used for epidemiological surveillance, outbreak detection, and evolutionary relatedness [37,78]. However, it is time-consuming, has high analytical costs, and requires a very high initial investment in hardware and software compared to PFGE [19,96,105].

#### 5.2.2. Outbreaks of *S. aureus* Bovine Mastitis

The usefulness of PFGE subtyping has been demonstrated in both the detection of outbreaks and surveillance in bovine mastitis [54,56,116] and food poisoning cases [25,59,117]. Table 3 shows the degree of clonal relationship between *S. aureus* strains and their sources and reservoirs of contamination, as identified by PFGE analysis. 

Several cases of *S. aureus* mastitis outbreak and surveillance have been reported worldwide. *S. aureus* clones that cause bovine mastitis are more likely to be unique to a herd [50,56,82,103,129,130]. However, the strains can spread between multiple herds or even wider geographic distribution areas [16,36,83,86,131]. In the works of Middleton et al. [101], Haveri et al. [82], Piccinini et al. [121], Capurro et al. [56], Mørk et al. [122], and da Costa et al. [120], *S. aureus* isolates from extramammary skin sites were found to be genotypically the same as isolates from milk. Teat skin, for some, and teat canals or hock skin, for others, were important potential reservoirs of *S. aureus* in herds with mastitis problems and explained cow-to-cow transmission. Teat skin contamination can be related to the persistent association between udder skin and bovine IMIs, according to the advice for more hygienic milking procedures [102]. Jørgensen et al. [83] found that isolates with band patterns similar to those of bulk milk were extracted from raw milk products and cases of mastitis, indicating the contaminating nature of *S. aureus* in infecting udders via bulk milk and then raw milk products. The studies of Monte et al. [112] and Lee et al. [16] revealed that clonally related MRSA were detected in milk samples, as well as in hand swabs in dairy herds or the environment, suggesting that manual milking could contribute to the spread of strains in the environment and to human skin. These findings suggest that the interface between animal populations and humans needs to be monitored for the early detection of changes in population dynamics [96]. Similar findings have been reported in the studies by Fagundes et al. [17], Pacha et al. [118], and Ronco et al. [132]. The predominance of identical clones within herds indicates that transmission between cows occurred from a common source. While a high degree of relatedness from strains to multiple herds suggests contagious transmission within and between farms, a greater variety of genotypes within herds is more likely related to environmental pathogens [133]. Therefore, *S. aureus* strains can be contagious or environmental, depending on their source, reservoir, and mode of transmission. Despite the large number of studies relating a high relationship between strains from one herd, it has also been shown that mastitis cases caused by multiple strains, phylogenetically distinct, may spread between animals and herds [10,54,97]. The presence of different sources of strains causing IMIs has been reported [13,35]. For example, in a study by Zadoks et al. [134], the cause of most cases of *S. aureus* mastitis was associated with strains adapted to the mammary gland, which were different from those found on the teat skin, indicating that not all infections were due to cow-to-cow transmission.

#### 5.2.3. Staphylococcal Food Poisoning Outbreaks

Staphylococcal food poisoning outbreaks (SFPOs) are considered one of the most common foodborne diseases throughout the world, affecting human health and food safety [117] and occurring typically after the consumption of foods contaminated with SEs [4]. Milk and its derivates represent important sources of foodborne pathogens [12]. They have been implicated in 5% of all staphylococcal foodborne outbreaks [135]. *S. aureus* can enter dairy food consumed by humans through various routes and which transmission has become a serious public health problem, as far as human, veterinary, and food safety is concerned. PFGE studies have investigated outbreaks and the surveillance of food poisoning in relation to the consumption of contaminated milk products elaborated by raw milks from bovine IMIs. These are awarded to raw cow cheese, potato mash made with raw milk, Chantilly cream desserts made with milk, or to the consumption of ultra-pasteurized milk (Table 3). André et al. [84] studied a dairy processing plant and established the relationship between *S. aureus* isolates from raw milk and from cheese, on one hand, and the difference between those from workers and those from cheese, on the other hand. The study demonstrated that raw milk is considered the probable source of contamination of cheese, exhibiting more diversity than hand workers strains. The same concern was highlighted in relation to raw milk cheeses by Arcuri et al. [123] and Aydin et al. [127], who reported that the high genotypic diversity of strains of *S. aureus* suggests numerous sources of contamination, including raw milk, processing environment, food handlers, and processing areas. As for Walcher et al. [136], PFGE profiles were found to be predominant among *S. aureus* isolated from both bulk milk samples and during the two cheese-making processes. In the study by Jørgensen et al. [116], isolates from mashed potatoes made with raw milk and isolates from bulk milk displayed indistinguishable banding patterns. As such, *S. aureus* in raw milk appeared to be the source of the outbreaks, and *S. aureus* in bulk milk was implicated in SFP related to raw milk-based foods. The study by Weiler et al. [126] reported strains isolated from three patients, one operator, and all types of milk samples. The authors showed an indistinguishable macrorestriction pattern, clearly indicating that milk handlers were most likely to be the source of the outbreak, since the food handlers, foods, and patients displayed the same *S. aureus* strains. This was in accordance with the findings of Ercoli et al. [125] and Gallina et al. [137].

#### 5.2.4. Others Specific Applications of PFGE Analysis

As the standard method for detecting outbreaks and conducting short-term surveillance in veterinary medicine, PFGE has significant applications in monitoring and control programs for bovine mastitis [10,65]. Other significant applications of PFGE typing include genetic diversity, transmission host [96] occurrence [17,129], and the persistence of specific genotypes isolates or clones [103,104,122], as well as determination of the dynamics of strain transmission [92] and tracking contamination [10].

## 6. WGS: New Future Approach as an Alternative to PFGE

The high resolution offered by WGS makes it suitable for phylogenetic studies and evolutionary relationships or transmission dynamics of different strains [32,37]. WGS is the process for constructing and determining the complete genomes of various bacterial pathogens [70]. This is made possible by next-generation sequencing (NGS), and, with these NGS platforms, clinical isolates can be compared to each other and to reference sequences over time and space with the precision of a single nucleotide difference [69]. Hundreds of gigabytes of data can be generated in a single experiment, providing unprecedented throughput and the ultimate resolution, resulting in an unambiguous relatedness of strains not just in terms of the global population structure but also in terms of local patterns of transmission [69,70]. For epidemiologic purposes, WGS is an extremely powerful and highly attractive tool in today’s control practices at the local, national, and international levels. However, it is only now beginning to be realized as a research tool for livestock animals. In cattle, WGS is becoming increasingly common for the study of IMIs associated with *S. aureus* isolates, as it is the preferred method for understanding phylogenetic relationships, evolution, and inter- and intraspecies differentiation, as well as the genetic basis of phenotypic characteristics, such as antibiotic susceptibility and virulence. Several studies have already shown that WGS based on either single-nucleotide polymorphisms (SNPs) [24,26,132] or on the gene-by-gene allelic profiling of core genome genes, frequently called core genome MLST (cgMLST) [24,75,78], represents the ultimate diagnostic typing tool that has been successfully applied for virulence factors, phylogenetic relationships, and the diversity of *S. aureus* in bovine milk. NGS/WGS outperform all other methods, such as PFGE, MLST, and *spa* typing, in terms of resolution, huge amounts of data, and discriminatory power, as well as affordability and throughput sequencing [24,74,78,96], although the cost-effectiveness of this technique is still an issue and its usefulness in veterinary epidemiology is still in its early stages.

## 7. Conclusions

Although different molecular techniques provide highly supported phylogenic data, PFGE remains, at this time, an appropriate tool for subtyping *S. aureus* isolates of bovine mammary origin. Since its epidemiological use requires interpretation criteria and resolution to distinguish clonally related isolates from unrelated isolates, PFGE patterns must always be interpreted in the context of the proper epidemiological and clinical characteristics of isolates.

The persistence of strains detected by PFGE coincides with herd problems, including increased bulk milk somatic cell counts, decreased milk production, antimicrobial resistance phenotypes, and the severity and chronicity of the symptoms. Other factors that are believed to influence the genetic diversity and that may contribute to the spread of the strains in the environment and humans include the herd size, animal traffic, hygienic milking procedures, and specific management practices. These associations support the need for the effective surveillance and treatment of *S aureus*-associated bovine IMIs.

There are also multiple practical problems with the use of PFGE that need to be addressed. This emphasizes the need to standardize the conditions to establish an accurate database with the possibility of interlaboratory comparisons and to develop an easy and rapid PFGE protocol that can analyze large numbers of samples, making it a reasonable option in the veterinary field.

## Figures and Tables

**Table 1:** The main characteristics of the types of instruments based on the principles of the PFGE procedure.

Types of PFGE	Comments	Discriminatory Power	Time Required	Cost	Ease of Use	References
CHEF	- Uses two homogeneous electric fields. - Resolves linear fragments of DNA. - Separates a very large number of DNA molecules into straight lanes; by 24 electrodes. - Orients the DNA over an angle fixed at 120°. - Requires 20–30 h of electrophoresis time.	Excellent resolution (Over 7000 kb)	Time consuming	High	Easy	[29,41,48]
FIGE	- Uses homogeneous electric fields and straight lanes. - Uses a conventional gel-electrophoresis chamber. - Orientates the DNA over an angle fixed at 180°. - Produces smaller fragments. - Requires 3–4 h of electrophoresis time. - Is less discriminating than CHEF.	Moderate resolution (Over 800 kb)	Rapid screening	Low	Easy and simple	[29,41,46,48]
TAFE	- Uses nonhomogeneous electric field and straight lanes in a vertical gel. - Results in different rates of DNA molecule migration. - Alternates electric fields oriented across the gel. - Orients the DNA over an angle varied from 115° to 165°.	Acceptable resolution (Up to 1600 kb)	Pratique	Low	Simple	[43,47,48]
OFAGE	- Uses two inhomogeneous electric field in two directions. - Resolves vertical alternating field electrophoresis. - Produces nonlinear and dissimilar electric fields. - Difficult comparison between the gel lanes. - Orients the DNA over an angle varied from 90° to 180°.	Good resolution (1000 kb–2000 kb)	Time consuming	High	Complex	[41,44,47,48]
RGE	- Produces straight lanes. - Uses a single homogeneous electric field. - Uses only one set of electrodes. - Orients the DNA over varied angles.	Good resolution (50–6000 kb)	Time consuming	Low	Easy	[41,45,48]
PACE	- Produces multiple, homogeneous electric fields and straight lanes. - Is better than FIGE and OFAGE.	Excellent Resolution (100 bp–6 Mb)	Time consuming	Very High	Complex	[41,44,48]

Abbreviations: CHEF: contour-clamped homogeneous electric field; FIGE: field inversion gel electrophoresis; OFAGE: orthogonal field-alternating gel electrophoresis; RGE: rotating gel electrophoresis; TAFE: transverse alternating field electrophoresis; PACE: programmable autonomously controlled electrodes; kb: kilobase fragments.

**Table 2:** The main characteristics of PFGE typing compared with the major molecular methods for *S. aureus* isolates.

Molecular Methods	Characteristics								
	Reproducibility	Typeability	Discriminatory Power	Ease of Use	Time Required	Interpretation	Cost Per Isolate	Throughput	Reference
PFGE	Goud	High *	Excellent	Difficult	Time- consuming (2–3 days)	Complex	Expensive (£4–7)	Low	[19,61,69]
MLVA	High	High	Good	Easy	Quick (24 h)	Complex	Inexpensive (£3–5)	High	[19,69]
MLST	High	High	Good	Difficult	Time- consuming (Days)	Simple	Very expensive (£20)	High	[19,69]
Spa	Excellent	High	Good	Easy	Quick (24 h)	Simple	Low (£3–5)	High	[19,69,114,115]
SCC*mec*	NA	Low	Good	Difficult	Time- consuming	Complex	High	High	[19,70]
WGS	High	Exceptional	Exceptional	Very Difficult	Time consuming	Complex	Very expensive £100	High	[19,69,105]
Microarray assay	High	High	Excellent	Difficult	Time consuming	Complex	High	High	[19]

Abbreviations: PFGE: pulsed-field gel electrophoresis; MLVA: multiple-locus variable number tandem repeat analysis; MLST: multi-locus sequence typing; spa: Staphylococcal protein A; SCCmec: Staphylococcal cassette chromosome *mec*; WGS: whole genome sequencing; NA: not available; * except MRSA isolates of ST398.

**Table 3:** The bovine IMIs related to environmental infections and food poisoning cases associated with the consumption of contaminated raw milk and dairy products analyzed by the PFGE method.

Origin of Samples	Epidemiological Investigations	Source of *S. aureus* IMIs or Food Poisoning	Country	Reference
Milk and hand swabs	Genotypic relatedness	Manual milking	Brazil	[112]
Bulk tank milk and milking equipment	Genotypic relatedness	Adherences on milking equipment	Chile	[118]
Cows, colostrum from heifers, heifer body sites, heifer environment, horn flies, and humans	Genotypic relatedness	Flies and heifer body sites	USA	[119]
Mastitic milk, extramammary sites (skin lesions) and human hands and nostrils	Genotypic relatedness	Teat’s skin	Finland	[82]
Milk of lactating cows, body sites, and environment of cows	Genotypic relatedness	Body samples (hock skin)	Sweden	[56]
Milk and teat skin swabs	Genotypic relatedness	Possibly teat’s skin	Brazil	[120]
Milk from cattle, heifers at the time of parturition, and cattle purchased for expansion	Genotypic relatedness	Teat and udder skin (Heifer body sites and lactating mammary gland)	USA	[101]
Curd, milk, and teat swab	Genotypic relatedness	Teat’s skin	Italy	[121]
Milk and teat skin	Genotypic relatedness	Milk and body sites	Norway	[122]
Individual milk and bulk milk from all lactating cows	Mastitis outbreaks	Milk and bodies	Japan	[54]
Mastitic cow’s milk, bulk tank milk, and Minas frescal cheese	SPOs surveillance	Raw milk, processing environment and food handlers	Brazil	[123]
Food handlers, raw milk, and Minas frescal cheese	SPOs surveillance	Raw milk contamination in cheeses	Brazil	[84]
Bulk tank milk samples and traditional cheeses	SPOs surveillance	Bulk tank milks	Austria	[124]
Milk of mastitic cows, bulk tank, and swabs from the environment	SPOs surveillance (Genetic relatedness)	Bulk tank milks, and raw milk products	Hungary	[91]
Infected udders, bulk milk, and raw milk products	Genotypic relatedness	Bulk milk	Norway	[83]
Raw milk (individual cows and bulk tank) and milking environment (equipment and milkers’ hands)	SPOs surveillance (Genetic relatedness)	Raw milk	Brazil	[16]
Milk (individual cows with subclinical mastitis) and bulk tanks	SFPOs Surveillance	Raw milk	Brazil	[17]
Raw cow milk	SFPOs surveillance	Raw cow milk with classical enterotoxin genes	Poland	[13]
Potato made with raw milk	SFPOs	Bulk milk	Norway	[116]
Cows, equipment, and environment processing Humans and cheeses	SFPOs	Raw milk products and Bulk milk	Norway	[83]
Different types of cheese	SFPOs	Raw milk	Romania	[59]
Chantilly cream dessert made with milk	SFPOs	Food (Chantilly cream) and Food handler	Italy	[125]
Strains of three patients, one operator, and all the milk samples	SFPOs	Milk handlers and operator	Paraguay	[126]
Raw milk and dairy products (cheese, butter, yoghurt, and cream)	SFPOs	Raw milk	Turkey	[127]
Cheese made from raw milk	SFPOs	Raw milk	France	[64]
Soft cheese made from unpasteurised cow milk	SFPOs	Milk cheese source of food poisoning	France	[128]
Cheese made from raw milk	SFPOs	Raw milk	France	[95]
Milkers’ hands	SFPOs surveillance	Farmers contaminating the milk	Brazil	[94]

Abbreviation: SFPOs: Staphylococcal food poisoning outbreaks.

## Data Availability

Not applicable.
